# Supplementary material for: A survey of human cancer-germline genes: Linking X chromosome localization, DNA methylation and sex-biased expression in early embryos
Source: PLoS Genet. 2025 Oct 15;21(10):e1011734. doi: 10.1371/journal.pgen.1011734 (PMC12527196; doi:10.1371/journal.pgen.1011734)
Supplement: S3 Table — (PDF) [file pgen.1011734.s006.pdf]

Table S3. List of datasets used in CTexploreR analyses

Type of data	Source	Accession code / File
RNASeq fastq files from normal tissues	ENCODE [77]	testis (ENCFF140UYT, ENCFF794EAB)<br>thyroid gland (ENCFF151GUG, ENCFF628TMU)<br>gastrocnemius medialis (ENCFF004CNM, ENCFF086LCO)<br>adrenal gland (ENCFF911BTP, ENCFF904VHM)<br>subcutaneous adipose tissue (ENCFF667CWY, ENCFF360KZB)<br>stomach (ENCFF741NGG, ENCFF582ILA)<br>upper lobe of left lung (ENCFF719YBM, ENCFF801ZKX)<br>suprapubic skin (ENCFF398KGB, ENCFF058JYK)<br>breast epithelium (ENCFF767QVV, ENCFF050GYP)<br>lower leg skin (ENCFF431RAQ, ENCFF008OVI)<br>sigmoid colon (ENCFF153ULW, ENCFF182OWD)<br>transverse colon (ENCFF411UIT, ENCFF992NAN)<br>spleen (ENCFF567ORO, ENCFF743QDT)<br>gastroesophageal sphincter (ENCFF250BPC, ENCFF842DCO)<br>tibial nerve (ENCFF534AYT, ENCFF935LBC)<br>esophagus muscularis mucosa (ENCFF091UZU, ENCFF163DLM)<br>omental fat pad (ENCFF745PTG, ENCFF824ZLA)<br>esophagus squamous epithelium (ENCFF585TOW, ENCFF230GLF)
RNA-Seq from cell lines treated with 5-Aza	ENCODE[77]	IMR5-75 CTL (SRR3326020/SRR3326021)<br>IMR5-75 DAC (SRR3326022/SRR3326023)<br>HCT116 CTL (SRR9108737/SRR9108738)<br>HCT116 DAC (SRR9108739/SRR9108740)<br>HEK293T CTL (SRR1618781/SRR1618782)<br>HEK293T DAC (SRR1618783/SRR1618784)<br>HMLER DAC (SRR3362409/SRR3362410)<br>HMLER CTL (SRR3362411/SRR3362412)<br>NCH612 CTL (SRR12105788/SRR12105789)<br>NCH612 DAC (SRR12105790/SRR12105791)<br>NCH1681 CTL (SRR12105792/SRR12105793)<br>NCH1681 DAC (SRR12105794/SRR12105795)<br>TS603 CTL (SRR12105780/SRR12105781)<br>TS603 DAC (SRR12105782/SRR12105783)<br>B2-1 CTL (SRR5363797/SRR5363798)<br>B2-1 DAC (SRR5363799/SRR5363800)
WGBS from healthy tissues	ENCODE[77]	adipose ENCFF318AMC<br>colon ENCFF157POM<br>oesophagus ENCFF625GVK<br>heart ENCFF536RSX<br>intestine ENCFF241AQC<br>lung ENCFF039JFT<br>muscle ENCFF121ZES<br>pancreas ENCFF763RUE<br>placenta ENCFF437OKM<br>skin ENCFF219GCQ<br>stomach ENCFF497YOO<br>testis ENCFF715DMX<br>thyroid ENCFF223LJW
